# Dogs (*Canis familiaris*) distinguish conspecific emotional chemosignals

**DOI:** 10.1038/s41598-026-41426-1

**Published:** 2026-02-26

**Authors:** Alice Wang, Alexandra Horowitz

**Affiliations:** 1 Richard Gilder Graduate School, American Museum of Natural History, 200 Central Park West, New York, NY 10024 USA; 2 Dog Cognition Lab, Department of Psychology, Barnard College, Columbia University, 3009 Broadway, New York, NY 10027 USA

**Keywords:** Dogs, Olfaction, Emotions, Animal cognition, Neuroscience, Psychology, Psychology, Zoology

## Abstract

Emotional communication facilitates social interactions among animals. While dogs can discriminate and exhibit context-appropriate behaviors in response to odor samples from stressed and non-stressed humans, their capacity to differentiate emotional odors from conspecifics—and the broader function of these cues in intraspecific communication—has not been investigated. Using a habituation-discrimination paradigm, 43 dogs were presented with odor samples from an unfamiliar dog collected after events marked as joyful, stressful, and baseline (relaxing). Subjects showed longer investigation time for novel versus repeated odors, indicating successful discrimination and dishabituation. Specifically, subjects distinguished between joy and baseline odors and between joy and stress odors. Additionally, dogs exhibited distinct behavioral responses depending on the emotional content of the odor, including differences in stress- and joy-related behaviors, proximity to targets (owner, stranger, and odor source), and body posture. Exposure to stress odors elicited the predicted attachment-related behaviors—characterized by increased proximity to their owners and reduced proximity to strangers—suggesting a response to conspecific emotional states that may reflect emotional contagion. These results demonstrate that dogs can perceive and behaviorally respond to emotional odors from conspecifics, underscoring the role of olfaction in canine intraspecific emotional communication.

## Introduction

Dogs (*Canis familiaris*) have been shown to detect and respond to emotional cues, primarily those related to human emotions^1–3^. This interspecific focus in canine communicative behavior reflects scientific interest in the evolution of communication between dogs and humans over the more than 10,000 years of domestication^4,5^. Throughout this shared evolutionary history, dogs have developed a unique role as human companions^6^, with no other domesticated species having evolved in such close proximity to humans^5^. This long-term cohabitation likely facilitated the development of dogs’ remarkable ability to functionally respond to human emotions through communicative cues^3^, including processing human emotional facial expressions^7^ and integrating these facial signals with directional gestures^8^. Moreover, dogs have shown lateralized brain patterns, cardiac activity, and behavioral responses indicating sensitivity to human emotional vocalizations^9^, and they look significantly longer at faces whose expressions align with the valence of concurrent vocalizations, thereby demonstrating their ability to integrate bimodal (visual and auditory) inputs to distinguish between positive and negative human emotions^10^.

Historically, research on canine communication has disproportionately emphasized vision and hearing—the dominant modalities in human communication—while largely overlooking olfaction^11^. Yet, olfaction is a primary and highly specialized sensory modality in dogs, playing a central role in their perception of the world^12^.

Recently, there has been a rise in canine olfactory research related to emotional communication, particularly on dog-human rather than conspecific (dog-dog) interactions^11^. In human-human communication, emotional states can be transmitted, in part, through odors^13^. Given the keen olfactory abilities of dogs, research has asked whether they can discriminate between human body odors collected at baseline and those obtained after a stress-inducing task, and dogs demonstrated an ability to “smell stress”^14^. Additionally, dogs exhibit behavioral responses consistent with human emotional chemosignals associated with fear and happiness^2^, and they adjust their perception of risk and reward in response to different human emotional chemosignals^15^.

Despite our growing understanding of how dogs perceive and process emotional content in human odors, few studies have explored how dogs respond to emotional content in conspecific odors. Olfaction has been shown to play a critical role in certain canine social interactions, such as the communication of reproductive status through female conspecific odors^16^. Given that dogs are recognized to experience emotions^17^ and possess the neuroanatomical structures necessary for basic emotional processing^18^, it is plausible that dogs also produce chemosignals linked to their emotional states and use them for intraspecific communication. Indeed, dogs exhibit a right-nostril bias when investigating conspecific anxiety odors^19^. Since the mammalian olfactory pathway primarily ascends ipsilaterally to the brain^20,21^, the observed right-nostril bias indicates activation of the dog brain’s right hemisphere, which is associated with processing arousal stimuli^22^. This pattern suggests that dogs likely perceive these odors as emotionally intense. However, it remains unclear whether dogs can discriminate between different emotional states in odors emitted by other dogs, and how such olfactory cues influence canine behavior beyond nostril lateralization and the presumed neural processing involved.

An established method for evaluating an animal’s ability to discriminate between odors is the habituation-discrimination paradigm. This approach has been used by Péron et al.^23^ to demonstrate that horses can differentiate between the individual body odors of unfamiliar conspecifics, and by Jardat et al.^24^ to show that horses can distinguish between human body odors associated with fear and joy contexts. In this paradigm, an individual is first repeatedly exposed to the same odor (in our study, a single, identical stimulus) across successive presentations (the habituation phase), followed by simultaneous presentation of the habituated odor alongside a novel odor (the discrimination phase). If dogs can distinguish between conspecific odors associated with two different emotional states, investigatory behavior should decrease across the habituation phase, followed by a significant increase in response to the novel emotional odor during the discrimination phase^23,24^. Furthermore, increased investigatory behavior toward the novel odor compared to the final odor presentation in the habituation phase would indicate dishabituation, further supporting the hypothesis that dogs can distinguish between the two odors^25–28^.

In addition to investigating dogs’ ability to discriminate emotional odors from conspecifics, the functional role of these olfactory cues remains unexplored. One potential function of emotional odor detection is the facilitation of emotional communication between conspecifics. The foundational mechanism underlying such communication is emotional contagion—the spontaneous transfer of emotional states between individuals—which may serve as a precursor to higher-order cognitive processes, such as theory of mind and perspective-taking^29^. Emotional contagion has been shown to confer evolutionary advantages in social species^30^ and is widely observed among conspecifics across various social animals^31^. In dogs, however, research on emotional contagion has primarily focused on interspecific interactions with humans, using visual^32^, vocal^1,33^, and olfactory^2^ modalities. Although some evidence suggests that dogs exhibit emotional contagion in response to conspecific auditory cues^1^, it remains unknown whether similar responses occur following exposure to conspecific-derived emotional odors. Addressing this gap will provide insight into the functional significance of detecting and responding to conspecific emotional odors in dogs.

D’Aniello et al.^2^ demonstrated that dogs exhibit emotional contagion in response to human emotional odors, offering a methodological framework for testing whether dogs also experience emotional contagion in response to conspecific emotional odors. They found that different human emotional odors induce systematically different behaviors in dogs, showing heightened interest in their owners when exposed to fearful human odors and heightened interest in strangers when exposed to happy ones. Such behaviors are thought to reflect the attachment system of dogs, given that dogs form bonds with their owners akin to the parent-offspring relationship in humans^34^ and exhibit behaviors that seek proximity to their caregivers in stressful situations^35–37^. Based on these results, we predict that if emotional states are transmitted between dogs through chemosignals in a manner similar to that between humans and dogs, stress odors from conspecifics will activate dogs’ attachment systems, prompting them to seek out their owners as a “secure base” and maintain closer proximity to their owners. We also predict that these dogs will respond to the stress odor with heightened stress-related behaviors. In contrast, exposure to joy odors from conspecifics is expected to increase the dogs’ confidence, making them more comfortable and interested in the environment and toward strangers, resulting in increased proximity to strangers. Finally, we anticipate that dogs exposed to the joy odor will exhibit more joy behaviors accordingly.

This present study investigates the role of olfaction in intraspecific communication in dogs, addressing the understudied contribution of the olfactory system and conspecific cues to canine social behavior. First, we used a habituation-discrimination paradigm to determine whether dogs can distinguish between body odors of an unfamiliar conspecific produced under conditions eliciting joy, stress, and baseline states. We then evaluated the behavioral response of subject dogs following exposure to these emotional odors in an open-space setting, with both their owner and a stranger present, to investigate whether emotional contagion occurs between dogs. By examining how dogs perceive and respond to conspecific emotional odors, this research aims to deepen our understanding of dogs’ core socio-cognitive abilities that may have facilitated domestication. Previous studies have shown that dogs’ social-communicative skills with humans were likely acquired during domestication. For example, dogs outperform wolves in using human social cues to locate hidden food in the object-choice paradigm, regardless of their age or rearing history with humans^38^. In the domain of emotional communication, while dogs exhibit more stress responses and vocalizations to human distress (e.g., crying) compared to emotionally neutral sounds (e.g., humming), pigs (*Sus scrofa domesticus*), another domesticated companion mammal, do not display similar reactions^39^. This finding suggests emotional contagion between dogs and humans may be specifically linked to dogs’ selection for cooperation during domestication rather than being a shared mammalian trait. While much research has focused on human-directed communication, such as detecting^14,19^ and reacting^2,15,40^ to human emotional chemosignals, this study explores whether similar abilities exist in dog-to-dog communication, which could have preceded domestication and laid the behavioral foundation for interpreting human emotional signals. In addition to advancing knowledge of social interactions between dogs, this research can lead to practical implications for improving canine welfare. By addressing how conspecific chemosignal cues may affect dog behavior, even in the absence of the original canine odor source, this study opens new avenues for investigating the impact of conspecific odors on dog behavior in environments with multiple dogs, such as shelters, multi-dog households, training facilities, and veterinary clinics.

## Methods

### Ethical approval

This research was approved by the Institutional Animal Care and Use Committee (IACUC) at Columbia University (protocol number: AC-AABZ3660) and the Institutional Review Board (IRB) at Barnard College. All experiments followed the study protocol approved by these committees and were performed in accordance with federal guidelines and regulations in the United States. This study is reported in accordance with ARRIVE guidelines. Participation by canine subjects was voluntary, as interpreted by their continued engagement in experimental tasks. The study was discontinued if a dog persistently did not engage with the tasks, displayed excessive destructive behavior toward the equipment, or if the owner requested termination. We obtained informed consent from subjects’ owners, and they were informed about their right to withdraw from participation at any time.

### Subjects

Pet dogs and their owners were recruited through the Barnard Dog Cognition Lab mailing list. A total of 44 dogs were initially recruited, of which 43 dogs (23 M, 20 F) participated in at least one phase of the study and had a mean age of around 5.3 years (± 40.1 months; range: 10 mo to 13 year 10 mo). Seventeen dogs were identified by their owners as purebred, while 26 were mixed-breeds (for details on the specific breeds, see Data Availability). All dogs were neutered or spayed, with the exception of two male dogs. Prior to recruitment, owners completed a questionnaire addressing inclusion criteria, confirming that their dog (1) has lived in their current home for at least 6 months, (2) has up-to-date vaccinations, (3) is comfortable in new situations and with new people, (4) is treat-motivated for engagement during the study, (5) is familiar with smelling conspecific scents (as confirmed by owners that they do not discourage sniffing objects or other dogs during walks), and (6) has no medical conditions or impairments affecting olfactory function. No subject dogs shared the same owner, so each owner participated in the study only once.

### Odor Stimuli

**Fig. 1:**
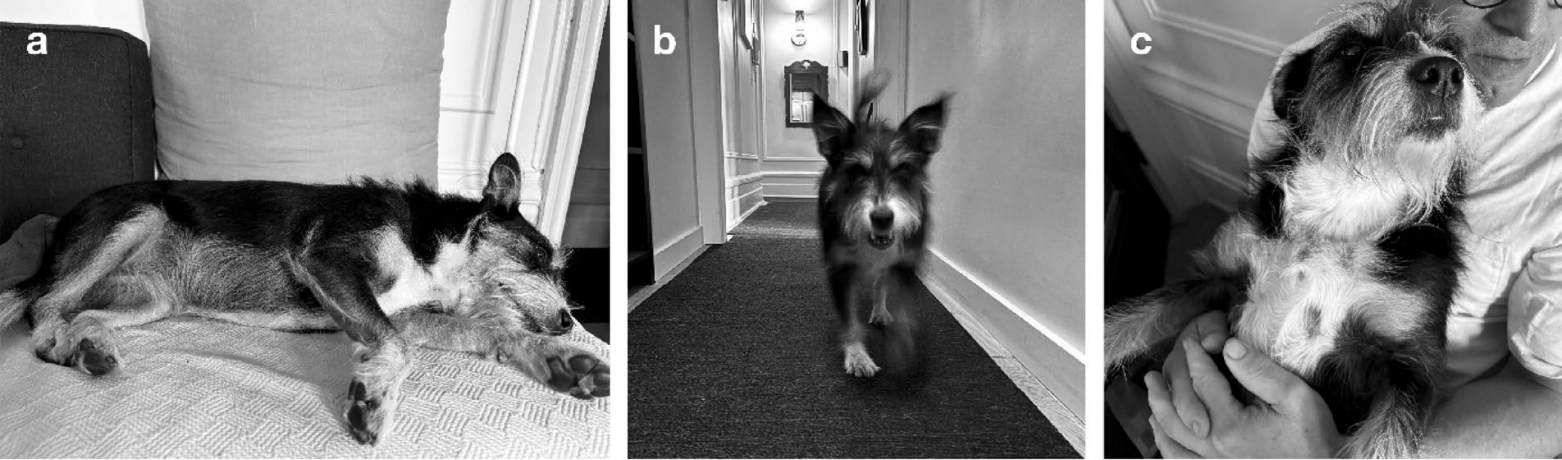
Visual representations of the donor dog during the three experimental conditions used to elicit the conspecific emotional odor stimuli: **(a)** “Baseline,” light resting while alone; **(b)** “Joy,” a 2-minute play session involving ball retrieval; and **(c)** “Stress,” a 2-minute nail-trimming procedure.

A 5-year-old spayed female mixed-breed dog, unfamiliar to the tested subjects, served as the donor for conspecific emotional odors. Odor samples were collected weekly over the four-month study period (April 2024, November 2024, February–March 2025) during three emotion-inducing events intended to elicit “joy”, “stress”, and “baseline”. The presumed emotional states were characterized as and collected in the order that follows: (1) “Baseline” was represented by the dog resting (lightly sleeping or going in and out of sleep) for several minutes by themselves (Fig. 1a), not in contact with other dogs or humans to minimize socio-positive interactions (e.g., cuddling) that could elevate oxytocin levels and potentially compromise the neutrality of the baseline odor for the purposes of this study^41^; (2) “Joy” emerged from a 2-minute play session, during which the dog repeatedly retrieved a ball tossed by the owner (Fig. 1b); and (3) “Stress” was induced through a 2-minute nail-trimming procedure, wherein the dog was restrained by an assistant while the owner trimmed the nails (Fig. 1c). The owner verified that the behavioral patterns observed during the emotional events were consistent with the intended emotional states. The donor dog exhibited behaviors indicative of joy, such as a relaxed open mouth (“play face”) and tail wagging during the “joy” event (Fig. 1b), and behaviors indicative of stress, such as flattened ears and attempts to escape restraint during the “stress” event (Fig. 1c). Similar behaviors were used to code joy and stress responses in the tested dogs later in the study (see “Data Analysis”).

The body odors were collected by the dog’s owner, wearing gloves, only when the dog was healthy and had not eaten for at least 2 h. After each emotional event, odors were collected using one side of one sterile cotton pad and gently rubbing against the inside of the dogs’ cheeks and gums (saliva samples) and the pads of one foot (interdigital secretions) for 5 s each. The same cotton pad was then flipped and rubbed against the area on either side of the anus (perianal secretions) for 5 s. Regarding the fixed order of sample collection, baseline was collected first to ensure the dog was resting prior to odor collection, as the collection procedure might otherwise disturb the animal. Stress was collected last, as stress-related hormones can remain elevated for extended periods^42^. To control for the effects of circadian rhythm on stress levels in dogs, which fluctuate throughout the day^43^, the three emotional odor samples were consistently collected within the same 2-hour morning window, starting at 10:00 a.m., for each batch. This approach ensured that all emotional conditions within a batch were collected within the same time frame and thus were frozen for an identical period, maintaining consistency across trial conditions.

The cotton pads were prepared for storage and processed for experimental use following a standardized protocol and under controlled conditions. After collection, each cotton pad was cut into quarters and stored in a sterile, labeled Ziploc bag at −20 °C. At least 30 min prior to each day’s experiments, the samples—each representing a different emotional event —were defrosted to room temperature. Following Jardat et al.^24^, the samples were then placed under a heating lamp to activate the odorants 5 min before the study began. The odor samples were placed into identical 3.3-cm wide round tin canisters with three air holes in the lids, and each canister was placed in a box (25 × 20 × 7.6 cm). The three boxes were identical, but labeled on the bottom, to ensure that the experimenter presenting the stimuli remained blind to the condition. After each day of experiments, the odor samples were re-stored in the freezer, having spent no more than 2 h and 15 min at room temperature. Each sample was used for a maximum of 2 days, with no more than two subjects participating per day. A previous study found no significant degradation of human saliva cortisol samples stored at − 18 °C and room temperature for up to 72 h, including after multiple thawing-freezing cycles^44^. Additionally, Siniscalchi et al.^19^ re-stored and used canine odor samples up to ten times, with results indicating that dogs were able to detect odors. Based on these findings, we adopted a protocol in which samples were exposed to room temperature for no more than 4 h and 30 min—well below the 72-hour threshold—and used far fewer than ten times to prevent scent degradation.

### Testing Room

**Fig. 2:**
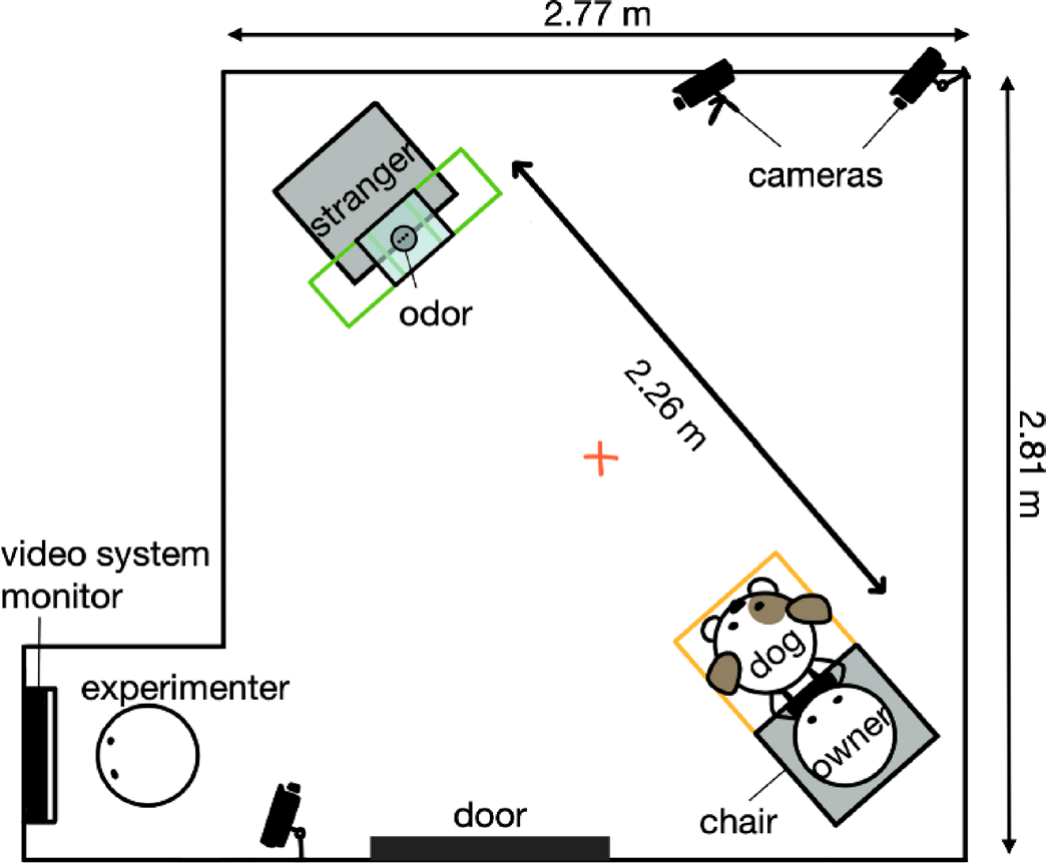
Layout of the testing room. During habituation-discrimination trials, the dog starts in the orange-taped area with the owner seated. The box position during habituation (single-box) is shown; green rectangles mark box placements during discrimination (dual-box), 20 cm apart. The red “X” indicates where the owner stands in Part 1 and where the odor box is located in Part 2, equidistant (1.13 m away) from the owner and stranger (an unfamiliar experimenter). Camera placement, doorway, and experimenter’s monitoring location are also shown.

All trials were conducted in the Dog Cognition Lab, in one of two similarly designed rooms, each with a single door and no windows (Fig. 2). Thirty-one subjects participated in experiments conducted in the first lab room, measuring 3.35 × 3.53 m. To identify potential sources of unintended odors in the environment^11^, we observed that a single ceiling vent, which provided heat during the winter, was the only airflow source. Twelve subjects participated in experiments in the second lab room, measuring 2.77 × 2.81 m, where no airflow sources were identified. The use of two lab rooms resulted from the relocation of the lab group during the study. The experimental design remained consistent across both rooms, and potential room-related variability was assessed and determined to be non-confounding in subsequent statistical analyses (see “Data Analysis” and “Results”). In both rooms, the floor was cleaned with a 70% isopropyl alcohol solution between subjects to minimize the transfer of scent information that could influence the dogs’ interactions with the presented odors. Subjects had access to a water bowl throughout the study. The dogs were never separated from their owner, and at the conclusion of the study, all dogs received a toy reward and a graduation certificate.

### Experimental Design

Owners brought their dogs to the Dog Cognition Lab on Barnard College’s campus in New York City, completed a consent form, and received instructions regarding their roles in the study, as detailed below. Data collection took place in April 2024, November 2024, and February–March 2025. Throughout the study, the owners accompanied their dogs to promote more naturalistic behavior in a novel environment^45^. Participants were scheduled to arrive in sequence, so no dogs nor owners interacted with, nor transferred information to, other participants prior to or following their own trial. Trials lasted for a maximum of 45 min and occurred between 5:30 p.m. and 7:30 p.m. Dogs were allowed to habituate to the room off-leash for 5 min or until they were visibly calm. Owners were unaware of the types of odors in the canisters, and experimenters described them simply as “natural odors” if asked. Experimenters instructed owners to allow their dogs to act “naturally,” emphasizing that there were no “right or wrong” behaviors.

#### Part 1: Habituation-discrimination paradigm

To test whether subject dogs could distinguish between joy, stress, and baseline emotional states in conspecific odors, each dog participated in three trials of habituation-discrimination tests, with all trials following a standardized procedure for odor presentations. In each trial, the dog was tested with one of three odor pairings—stress vs. joy, joy vs. baseline, or baseline vs. stress—assigned in a randomized order. An inter-trial interval of 1 min separated each trial. Experimenters instructed owners to remain seated with their dog on-leash at their feet at the start of every presentation. To encourage the dogs to approach the boxes throughout this study, at the start of each trial, the first box presented was a box with a treat inside it (“treat-box presentation”). The experimenter placed the box on the designated spot on the floor, 2.26 m from the owner’s chair, and knelt behind it (Fig. 2). Opening the lid, the experimenter alternated glances between the box and the dog while stating, “Hi puppy, what’s this?” This phrase was repeated two additional times in a high-pitched voice characteristic of dog-directed speech^46^, with the experimenter tapping the sides of the box with each utterance. The experimenter then rose and moved to the corner, turning her back to the owner and dog. This action and a verbal “okay” signaled to the owner, as pre-instructed, that they could stand up and step forward toward the stimulus, positioning themselves on a designated spot (Fig. 2). While standing by the box, the first 14 owners were instructed not to engage or signal their dogs in any way. To enhance participant cooperation and minimize the need for frequent reminders, we developed and implemented an owner-distraction task for the remaining 29 owners. Owners were asked to focus on reading a poem on the wall, so as to distract them from observing and potentially influencing their dog’s behavior. Any potential variability resulting from the introduction of the owner-distraction task was assessed and determined to be non-confounding in subsequent statistical analyses (see “Data Analysis” and “Results”). Owner behavior was monitored using a video system, and corrections were made regarding their positioning or gaze direction as needed. The experimenter used a mirror or the video camera system to monitor when the dog consumed the treat. Upon consumption, the experimenter turned to retrieve the box, concluding the treat-box presentation and beginning the emotional odor trials via the habituation-discrimination paradigm. If the dog did not eat the treat within 30 s, the treat box was presented again with a new treat. Following a second presentation, emotional odor trials began if the dog (1) consumed the treat, (2) investigated the treat without consuming it, or (3) failed to approach the box after three presentation attempts.

Emotional odor trials followed the habituation-discrimination paradigm and consisted of two phases: a “habituation” phase and a “discrimination” phase. In the habituation phase, the experimenter presented an identical stimulus—body odor from a single emotional condition (e.g., the joy odor)—to the subject in three consecutive, single-box presentations. Following this, in the discrimination phase, the experimenter presented two boxes—both the habituated odor (e.g., the joy odor) and a novel odor associated with a different emotional condition (e.g., the stress odor)—side-by-side, 20 cm apart. To account for potential side bias^47^, the left and right positions of the original and novel odors were randomized. Experimenters presenting the stimulus boxes adhered to the same script and protocol as the treat-box presentation, with the modification that each presentation ended either after a minimum of 20 s or when the dog stopped investigating the box, whichever duration was longer. “Investigation” was defined as the dog’s nose being within 10 cm of the box^48,49^. All trials were videorecorded for subsequent coding of the dog’s investigation duration directed toward the stimulus boxes (see “Data Analysis”).

We implemented an “owner-enhanced” protocol whenever subject dogs failed to approach the stimulus box(es) within 20 s during the first presentation of the habituation phase or during the discrimination phase. If the dog did not approach the stimulus during the first habituation presentation, the experimenter followed the standard procedure; however, upon turning away and saying “okay,” the owner was instructed to drop the leash, walk to the experimenter’s presentation position, kneel, and hold the odor canister at the dog’s eye level while maintaining eye contact with the canister. The presentation ended either after a minimum of 20 s or when the dog stopped investigating the canister, whichever duration was longer. Three dogs required this procedure during the first habituation presentations of the joy and stress odor conditions, and four dogs during the baseline condition. If the dog did not approach either stimulus during the discrimination phase, the procedure mirrored that of the owner-enhanced habituation presentation. The owner held both canisters above the respective stimulus boxes at their dog’s eye level, while focusing their gaze on the floor between the canisters to avoid inducing a side bias. If the dog still failed to approach the boxes, the next odor combination was introduced, starting with the treat-box presentation. During the discrimination phase, six dogs required the owner-enhanced procedure for the stress-baseline and joy-baseline conditions, and seven dogs for the joy-stress condition.

#### Part 2: Testing behavioral reactivity to conspecific emotional odors

Twenty-four of the 43 dogs participated in Part 2 of the study, which used a between-subjects design to gauge their behavior after exposure to an odor sample representing different emotional conditions, in the company of their owner and a stranger. Each dog was randomly assigned to one of three stimulus odor conditions: joy, stress, or blank (a cotton pad with no odor added). Nine dogs (4 males, 5 females) were exposed to the joy condition, nine (5 males, 4 females) to the stress condition, and six (3 males, 3 females) to the blank condition. Two additional dogs were included in Part 2 but did not investigate the odor within the allotted time frame and thus were excluded from analyses. Three dogs discontinued their trials before reaching Part 2, and fourteen dogs were unable to participate due to the deferred implementation of this portion of the study. This section of the study occurred after a 1-minute break following Part 1. In Part 2, dogs were off leash, and a second seat was placed 2.26 m from the owner’s seat (Fig. 2). The experimenter presented a box with one stimulus odor, using the presentation method from Part 1, and placed the box equidistant between the seats (Fig. 2). The owner was instructed to walk behind the box, kneel, focus their gaze on the canister, and hold up the canister to the dog’s eye-level. This presentation ended after a minimum of 20 s or when the dog stopped investigating the canister—whichever duration was longer—as directed by the experimenter monitoring the dog using a mirror or the video camera system. Afterward, the owner returned to their seat. The owner and stranger (a second experimenter not previously involved in the presentations during Part 1) then sat in their seats on opposite sides of the lab, equidistant from the odor box. Both the owner and the stranger were blinded to the condition and given a form to fill out so as to not draw the attention of the dog or respond to any dog solicitations. The dog was permitted to move freely around the room. Each trial ended either 1 min after both individuals were seated or, if the dog investigated the odor box for the first time only after they were seated, 1 min following this initial investigation. Each trial was video recorded for later coding of the frequency of various behaviors (see “Data Analysis”).

### Data analysis

For Part 1, frame-by-frame video playback in BARC v.1.0.0 (https://github.com/emilydringel/BehavioralCodingSoftware*)* was used to code investigation time in seconds. Investigation time was defined as when the dog’s nose was within 10 cm (4 in.) of and oriented toward the box^48,49^. Each investigative phase ended when the dog’s nose moved beyond 10 cm from the box or was no longer directed toward it. Investigation time was cumulatively recorded for the duration of the stimulus presentation, with coding concluding when the experimenter turned around and retrieved the stimulus box. The coder was blind to the emotional condition of the odors presented.

The Wilcoxon signed-rank test was used to compare investigation durations to evaluate three behavioral phenomena: (1) habituation, assessed by comparing investigation time between the first and third presentations of the repeated odor; (2) dishabituation, assessed by comparing investigation time of the third presentation of the repeated odor to that of the novel odor; and (3) discrimination, assessed by comparing investigation time of the fourth presentation of the repeated odor to that of the novel odor. The investigation time during the third presentation of the repeated odor was compared to the fourth presentation to assess potential dishabituation resulting from the change in presentation format (i.e., from a single box to two boxes). To examine possible side bias during the discrimination phase^47^, linear regression analyses were conducted separately for each emotional pairing, with the side of the novel odor (left/right) as a predictor of the difference in investigation duration between the novel odor and the third or fourth presentation of the habituated odor. Given that the owner distraction task was implemented in November 2024 and February–March 2025 but not in April 2024, and that testing occurred in different rooms between April and November 2024 vs. February–March 2025, the time period of data collection was also included as a potential confounding variable in the regression models.

For Part 2, point-sampling was used to assess the behavioral responses during the 1-minute trial. The frequency of stress and joy behaviors (Table 1) was recorded every 5 s, resulting in 12 sample points per dog. Each sample point captured all behaviors exhibited during the 5 s leading up to that point (following D’Aniello et al.^2^). To assess proximity as a proxy for the attachment behaviors considered in D’Aniello et al.^2^, we recorded the dog’s location relative to three targets: the owner, the box containing the odor, and the stranger (i.e., the unfamiliar experimenter). At each 5-second interval, the target to which the dog was closest to—and at least within one body length of—received a score of 1, while all other targets received a score of 0. This measure was based on the dog’s closest body part (e.g., nose, head, paw, tail) to each target during the 5-second window preceding the sample point. The dog could be most proximate to multiple targets in one sample. During video analysis, distinct postures were observed across trials, which prompted further investigation. Posture was recorded at the end of each 5-second interval, with the possible postures being upright (standing on their feet), sitting (hindquarters down and forequarters elevated), and prone (lying on the stomach). A score of 1 was assigned to the posture the dog assumed, and a score of 0 was given to the other two postures. The subject could not assume more than one posture in each sample. As in Part 1, the coder was blind to the emotional condition of the odors presented.

These behavioral data were averaged across the dogs for each sample point in each odor condition. Comparisons of all behavioral data were made between conditions, incorporating all 12 sample points for each condition. The normality of the data was assessed using the Shapiro-Wilk test. If the data were normally distributed, ANOVA with Tukey’s pairwise post hoc comparisons was used. In cases where the data deviated from normality, a Kruskal-Wallis test with Dunn’s post-hoc comparisons was used. To determine how the interaction between sex and emotional odor condition variables affect dogs’ behavioral responses, specifically regarding the frequency of stress and joy behaviors and proximity, we used a generalized linear model (GLM) with Gaussian distributions. Any unreported comparisons showed no statistical significance (*p* > 0.05). All statistical analyses were performed with RStudio 2023.06.2 + 561.

## Results

### Habituation and discrimination to emotional odors

**Fig. 3:**
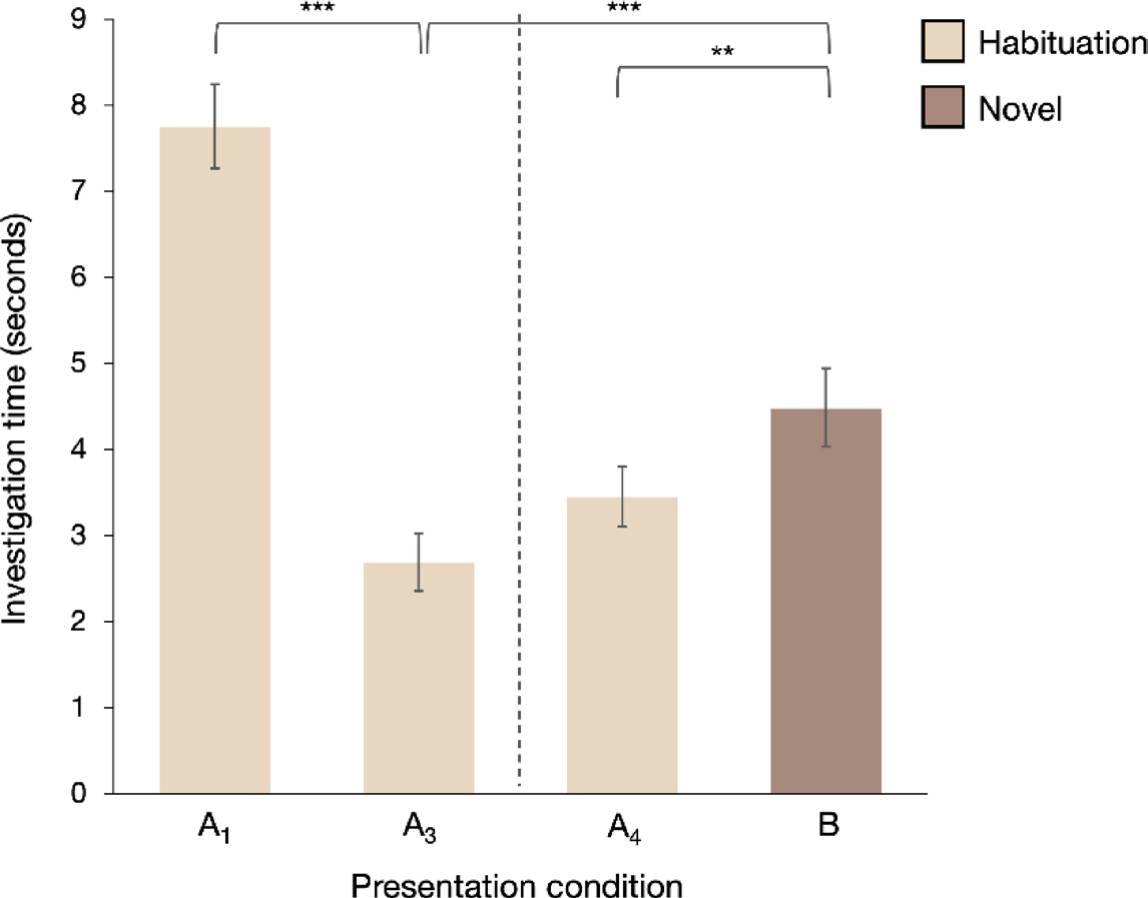
Investigation time for habituation, discrimination, and dishabituation trials across all emotional odor conditions. Investigation time decreases from the first (A_1_) to the third (A_3_) presentation of the habituation odor (A) across all trials, demonstrating habituation to the odors. Investigation time is significantly greater for the novel odor (B) compared to the fourth presentation of the habituation odor (A_4_), indicating successful discrimination between the odors. Additionally, investigation time for the novel odor (B) is greater than for the third presentation of the habituation odor (A_3_), demonstrating dishabituation. Data presented as means ± S.E.M. ***p < 0.001, **p < 0.01.

When data from all odor pairings were pooled, a Wilcoxon signed-rank test comparing the first and third odor presentations across all emotional conditions (joy, *n* = 40; stress, *n* = 41; baseline, *n* = 41) revealed a significant decrease in the dogs’ investigation time during the habituation phase (third presentation vs. first: *V* = 7108, *p* < 0.001), indicating successful habituation to the presented odor (Fig. 3). In the discrimination phase, a Wilcoxon signed-rank test showed that the dogs spent significantly more time investigating the novel odor compared to the fourth presentation of the habituated odor (*V* = 2704, *p* = 0.008), which was presented simultaneously, indicating discrimination between the two odors (Fig. 3). Additionally, the dogs spent significantly more time investigating the novel odor than the third presentation of the habituated odor (*V* = 2061.5, *p* < 0.001), which was presented prior to the introduction of the novel box, suggesting dishabituation to the novel odor (Fig. 3).

**Fig. 4:**
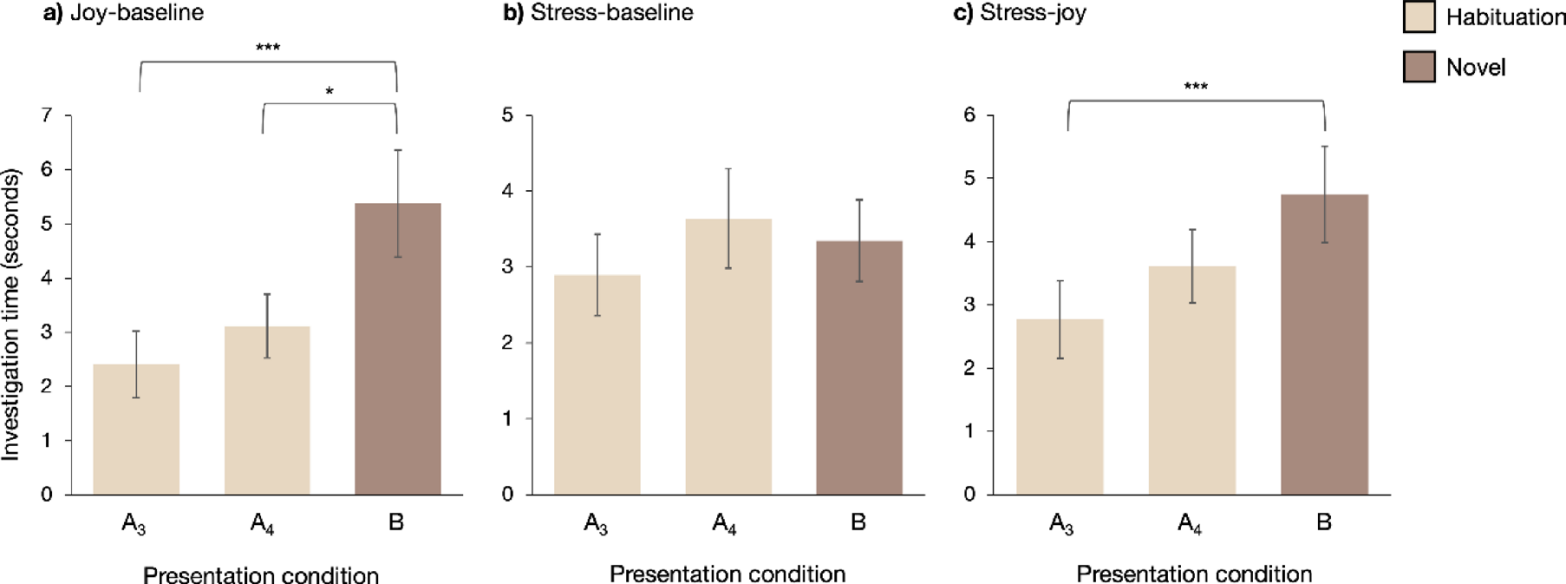
Investigation time for habituation, discrimination, and dishabituation trials across the three emotional odor pairings. A_3_ and A_4_ represent the third and fourth presentations of the habituation odor (A), respectively, while B denotes the novel odor. **(a)** Discrimination (A_4_ vs. B) and dishabituation (A_3_ vs. B) observed in the joy-baseline comparison. **(b)** No evidence of discrimination or dishabituation in the stress-baseline comparison. **(c)** Dishabituation without discrimination in the stress-joy comparison. Data presented as means ± S.E.M. ***p < 0.001, *p < 0.05.

**Fig. 5:**
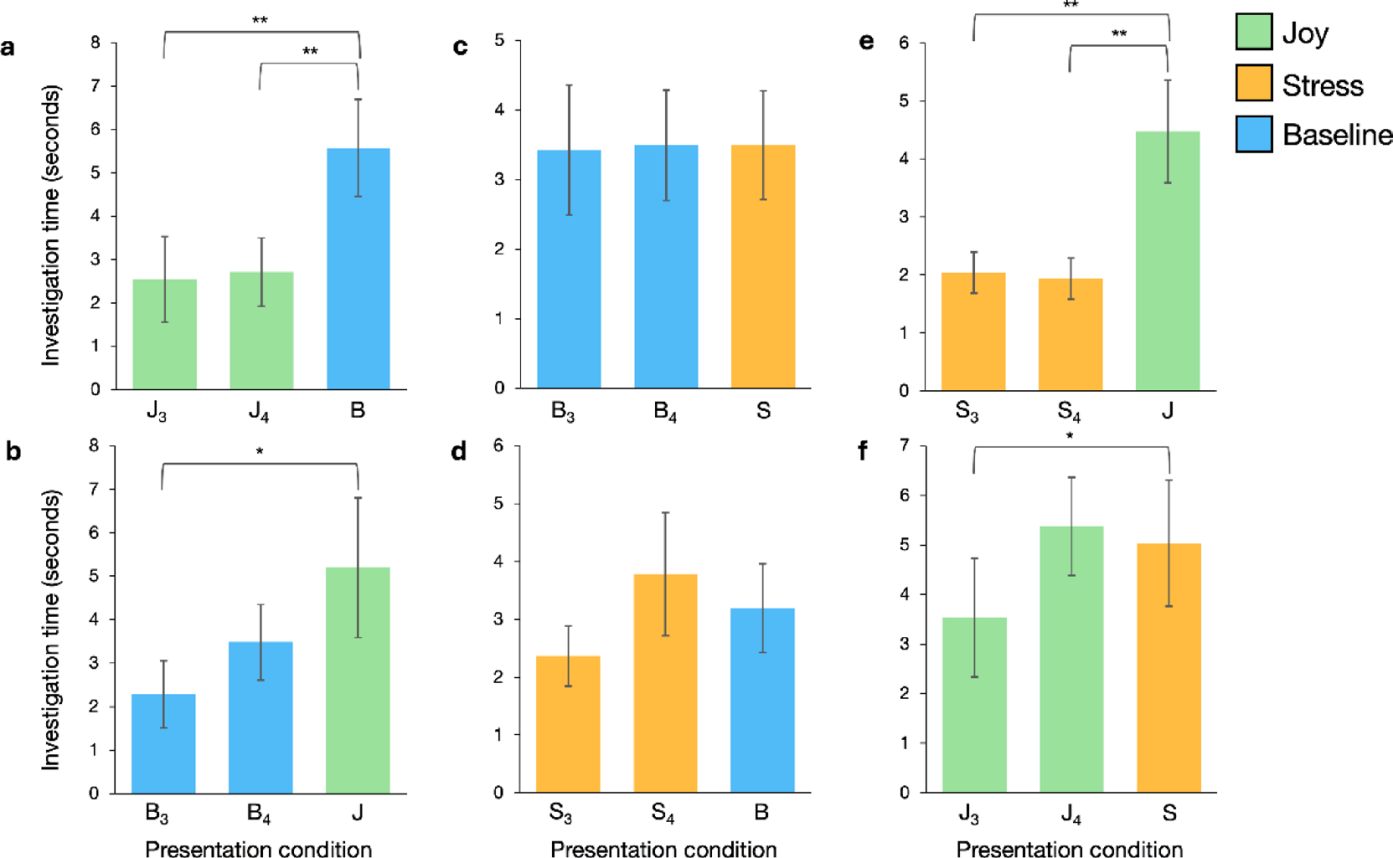
Investigation time for habituation, discrimination, and dishabituation trials across the six emotional odor presentation combinations. The first two bars in each graph represent the third and fourth habituation odor presentations (denoted as letter subscript 3 and 4, respectively), while the third bar (in a different color) indicates the novel odor. **(a)** Discrimination and dishabituation when joy (J, green) was the habituation odor and baseline (B, blue) was novel. **(b)** Dishabituation without discrimination when baseline was habituation and joy was novel. **(c)** No discrimination or dishabituation when baseline was habituation and stress (S, orange) was novel. **(d)** No discrimination or dishabituation when stress was habituation and baseline was novel. **(e)** Discrimination and dishabituation when stress was habituation and joy was novel. **(f)** Dishabituation without discrimination when joy was habituation and stress was novel. Data presented as means ± S.E.M. **p < 0.01, *p < 0.05.

Comparisons of specific odor pairings revealed that dogs investigated the novel odor significantly longer than the habituated odor only in the joy versus baseline condition (*n* = 40; novel vs. fourth habituation: *V* = 226, *p* = 0.013, indicating discrimination; novel vs. third habituation: *V* = 133, *p* < 0.001, indicating dishabituation; Fig. 4a). Dogs investigated the novel odor significantly longer than the fourth habituation presentation when joy was the habituated odor and baseline the novel odor (*V* = 26, *p* = 0.004; Fig. 5a), but not when baseline was habituated and joy was novel (*V* = 94, *p* = 0.470). However, in both cases, dogs investigated the novel odor significantly longer than the third habituation odor presentation (joy habituated, baseline novel: *V* = 25, *p* = 0.003; baseline habituated, joy novel: *V* = 44, *p* = 0.042; Fig. 5a, b), indicating that dogs discriminated between joy and baseline odors and dishabituated to the novel odor in this pairing.

No significant difference in investigation time was observed between stress and baseline (*n* = 41, *V* = 412, *p* = 0.820) (Figs. 4b and 5c and d) or between stress and joy (*n* = 41, *V* = 301, *p* = 0.095) (Fig. 4c) when comparing the fourth habituated odor presentation to the novel odor, with some caveats: When stress was the habituated odor and joy the novel odor, dogs investigated joy significantly longer (*V* = 23, *p* = 0.001; Fig. 5e). There was also a significant increase in investigation time for the novel odor when comparing the third presentation of the habituated odor to the novel odor for stress and joy (overall: *V* = 127, *p* < 0.001; stress habituated, joy novel: *V* = 29, *p* = 0.003; joy habituated, stress novel: *V* = 34, *p* = 0.045; Figs. 4c and 5e and f). Dogs dishabituated to the novel odor between stress and joy and discriminated between stress and joy odors when stress was the repeated odor and joy was the novel odor.

Dogs showed no evidence of format-induced dishabituation or side bias across conditions. In the overall analysis, we observed no significant differences in investigation time between the third and fourth presentations of the repeated odor, indicating no dishabituation due to the change in presentation format from one to two boxes (*V* = 2977, *p* = 0.087). Moreover, linear regression analyses revealed no statistically significant side bias or differences across data collection time periods (between April 2024, *n* = 14; November 2024, *n* = 17; and February–March 2025, *n* = 12) across all emotional pairings (*p* > 0.05).

### Stress and joy behaviors

**Fig. 6:**
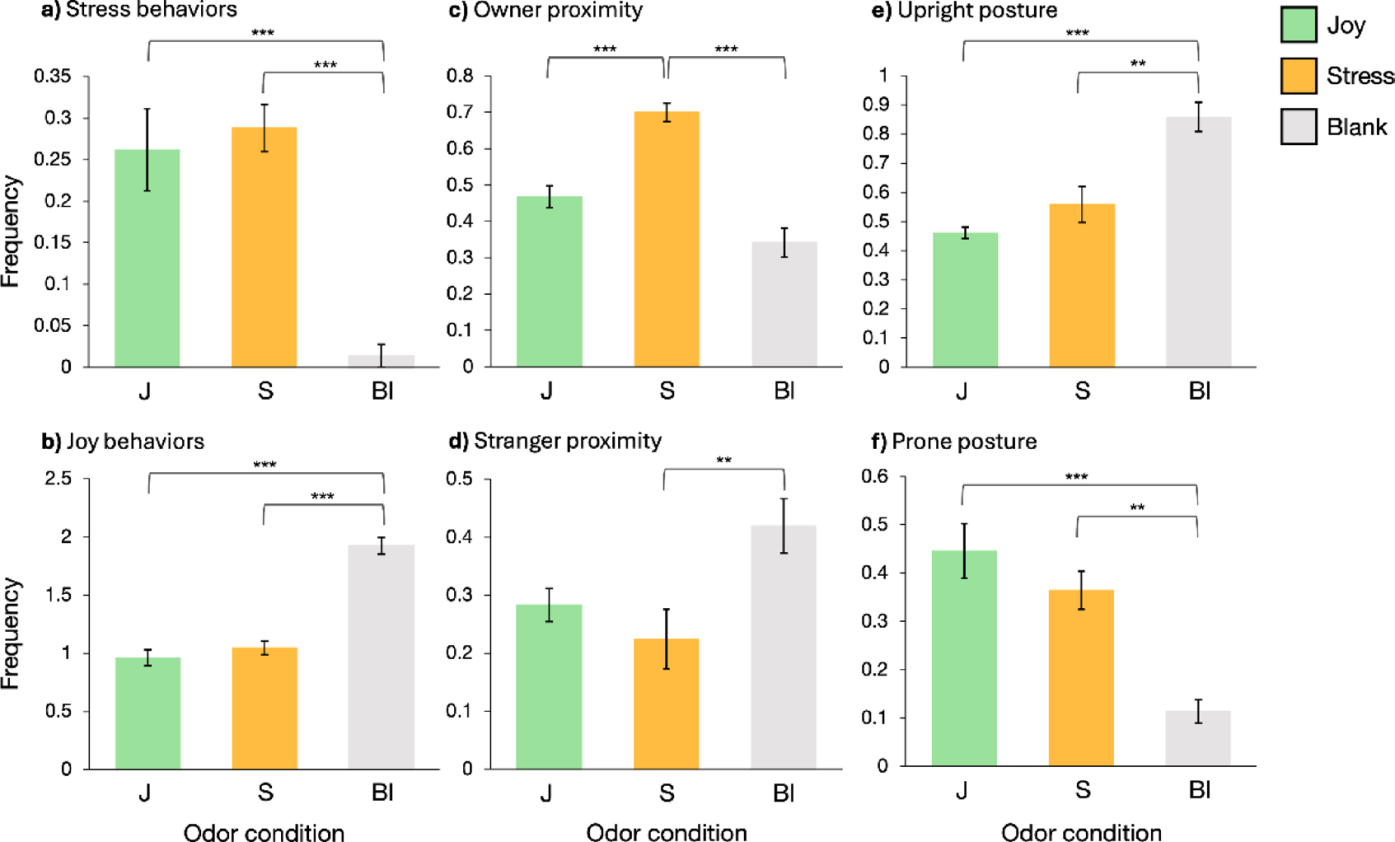
Behavioral frequencies during the 1-minute trial following exposure to joy (J, green), stress (S, orange), or blank (Bl, gray) odor conditions. Behavioral frequencies were cumulatively binned in 5-second intervals and averaged across dogs for each odor condition. **(a)** Fewer stress behaviors were observed in the blank condition compared to joy and stress odors. **(b)** Joy behaviors were more frequent in the blank condition than in the joy and stress conditions. **(c)** Stress odors elicited increased proximity to the owner relative to joy and blank conditions. **(d)** Stress odors resulted in reduced proximity to the stranger compared to the blank condition. **(e)** Upright postures were more frequent in the blank condition than in the joy and stress conditions. **(f)** Prone postures were less frequent in the blank condition than in the joy and stress conditions. Data presented as means of the 12 sample points ± S.E.M. ***p < 0.001, **p < 0.01.

The frequency of stress and joy behaviors exhibited by dogs across the 1-minute interval varied significantly depending on the emotional odor they were exposed to. Statistical analysis revealed a significant effect of odor condition on the frequency of stress behaviors (Kruskal-Wallis test: *H*(2) = 20.96, *p* < 0.001). Post-hoc comparisons indicated that dogs exhibited significantly fewer stress behaviors in the blank condition than in both the joy (Dunn’s post hoc: *p* < 0.001) and stress (Dunn’s post hoc: *p* < 0.001) conditions (Fig. 6a). Statistical analysis of joy behaviors showed a significant difference in frequency across conditions (Kruskal-Wallis test: *H*(2) = 23.98, *p* < 0.001). Post-hoc comparisons revealed that dogs displayed significantly more joy behaviors in the blank condition than in both the joy (Dunn’s post hoc: *p* < 0.001) and stress (Dunn’s post hoc: *p* < 0.001) conditions (Fig. 6b).

### Proximity

Subject dogs’ proximity to their owner and to a stranger differed across the emotional odor conditions. The frequency with which dogs remained closest to their owner varied significantly across the three odor conditions (Kruskal-Wallis test: *H*(2) = 23.11, *p* < 0.001). Post-hoc comparisons revealed that dogs were significantly more likely to be near their owner in the stress condition compared to both the blank (Dunn’s post hoc: *p* < 0.001) and joy (Dunn’s post hoc: *p* < 0.001) conditions (Fig. 6c). No significant differences were found in the frequency of dogs being closest to the odor box across conditions (ANOVA: *F* = 1.035, *p* = 0.370). Proximity to the stranger also varied by emotional odor condition (Kruskal-Wallis test: *H*(2) = 8.28, *p* = 0.016), with dogs in the stress condition being significantly less likely to be near the stranger compared to the blank condition (Dunn’s post hoc: *p* = 0.002) (Fig. 6d).

### Posture

Dogs displayed different postural behaviors across emotional odor conditions. The frequency of an upright posture varied significantly between conditions (Kruskal-Wallis test: *H*(2) = 16.89, *p* < 0.001). Specifically, post-hoc analysis indicated that dogs were more often standing in the blank condition compared to both the joy (Dunn’s post hoc: *p* < 0.001) and stress (Dunn’s post hoc: *p* = 0.004) conditions (Fig. 6e). No significant differences were found for the sitting posture across conditions (Kruskal-Wallis test: *H*(2) = 5.08, *p* = 0.079). However, a significant difference was found in the frequencies of dogs assuming the prone position (Kruskal-Wallis test: *H*(2) = 15.6326, *p* < 0.001), with dogs laying down less frequently in the blank condition compared to both the joy (Dunn’s post hoc: *p* < 0.001) and stress (Dunn’s post hoc: *p* = 0.005) conditions (Fig. 6f).

### Sex and emotional condition interactions

When exposed to the stress odor condition, females showed significantly higher frequencies of stress behaviors (AIC=−39.927, ß=−0.281, SE = 0.100, t=−2.796, *p* = 0.007) and significantly lower frequencies of joy behaviors compared to males (AIC = 45.290, ß=0.943, SE = 0.181, t = 5.201, *p* < 0.001). Females exhibited a higher frequency of proximity to their owner under the stress condition compared to males (AIC=−42.484, ß=−0.300, SE = 0.099, t=−3.044, *p* = 0.003). In contrast, males showed a significantly higher frequency of proximity to the stranger when exposed to the joy condition, compared to females (AIC=−11.885, ß=0.339, SE = 0.122, t = 2.780, *p* = 0.007).

## Discussion

Dogs in this study demonstrated the ability to distinguish between the body odor of a conspecific gathered under different emotion-eliciting conditions, as evidenced by longer investigation durations for novel compared to repeated odors. Specifically, dogs were able to distinguish joy odor from baseline odor, and joy odor from stress odor, through either odor discrimination or dishabituation, or both. Yet, subject dogs did not distinguish between stress and baseline odors using either discrimination nor dishabituation. This finding is unexpected in light of prior human research, which suggests that odors of negatively valenced emotional states—such as fear—are typically more distinguishable from odors of neutral states than are odors linked to positively valenced emotional states^50^. Given dogs’ heightened olfactory sensitivity relative to humans^12^, it would be reasonable to expect that conspecific stress odors are more readily distinguishable from a baseline state than joy-related odors are. This expectation is further supported by previous findings indicating that dogs can discriminate between human-derived stress and baseline odors^14^. Contrary to this prediction, our results revealed the opposite pattern: dogs more reliably differentiated conspecific joy odors from baseline, while stress odors were not significantly distinguished from baseline.

One possible explanation for the lack of differentiation between stress and baseline odors is that both may have inadvertently represented stressful states in our study. In particular, the baseline samples were collected during the rest-to-wake transition, since the sampled dog was inevitably awakened by the odor collection method. This interruption of rest for odor collection may have induced mild stress, akin to the cortisol awakening response observed in humans^51^, which could have influenced the baseline odor profile to mirror that of a stress odor. This procedural factor might have diminished the chemical distinction between the two odor conditions. Alternatively, it is possible that the odor representing the stress condition was not collected following a sufficiently stress-inducing event, thus leading to an odor profile more similar to baseline. However, this explanation seems less plausible given the results from Part 2 of this study, which demonstrated that conspecific stress odors elicited social behavioral responses characteristic of stress in dogs. A third potential explanation is that dogs did distinguish between stress and baseline odors, but the method of measuring investigation duration was not an ideal indicator. The novelty of the stress odor might not have resulted in longer investigation durations, as stress-related odors may also have been aversive. Dogs may have been less motivated to spend time near a stress-inducing stimulus, leading to an intermediate investigation duration: not shorter than baseline because the odor was novel, but not longer because it was off-putting. Findings from Part 2 support the notion that dogs do indeed perceive conspecific stress odors as distinct from other emotional odors.

In Part 2, consistent with our hypothesis, dogs adjusted their attachment behavior based on the emotional valence of the olfactory cues. Exposure to conspecific stress odors was associated with increased proximity to the owner relative to both the joy and blank conditions, a pattern consistent with the secure base effect^37^. This finding aligns with previous research by D’Aniello et al.^2^, which reported that dogs exhibited increased frequency and duration of owner-directed behaviors following exposure to human-derived fear chemosignals, underscoring the involvement of olfaction in interspecific emotional transfer. Our results extend this phenomenon to intraspecific contexts, demonstrating that similarly negatively valenced olfactory cues from conspecifics can likewise elicit security-seeking responses. Additionally, exposure to stress odors in conspecifics was linked to decreased proximity to strangers compared to the blank condition, suggesting that stress olfactory cues may also promote social caution toward strangers. Collectively, these behavioral adjustments highlight the functional role of emotional odors in shaping intraspecific social behavior in dogs, particularly under conditions of perceived stress.

Exposure to conspecific joy odors did not increase subject dogs’ confidence or social interest toward strangers as predicted. We originally hypothesized that emotional contagion would manifest an increased interest in a stranger—as operationalized by closer proximity—following exploration of joy odors, relative to both stress and blank conditions. However, this pattern was not observed in the current study. Emotional contagion in non-human animals has been more frequently documented in the context of negatively valenced emotions, such as stress or fear^31^, suggesting that the transmission of positively valenced emotions may be less robust or more difficult to detect through behavioral measures. It is important to note, though, that D’Aniello et al.^2^ reported increased stranger-directed behaviors in dogs following exposure to happiness odors from humans, suggesting potential differences in the transmission of positive emotional states in interspecific versus intraspecific contexts, or variations in the specific methods required to detect such transmission. Alternatively, it could be that non-human odors, such as the conspecific odors used in this study, do not influence dogs’ perception of humans in the same way that human odors do, where the odor source matches the object of perception. This interpretation suggests that emotional contagion through olfaction in dogs could be context-specific and manifest differently depending on the species involved. To test this hypothesis, future research would have to compare attachment behaviors in dogs exposed to either conspecific and human-derived odors, using consistent parameters (such as proximity, as used here, versus ethograms, as used by D’Aniello et al.^2^), and evaluate how the same dogs respond to odors from different species.

Both additional behavioral proxies—including measures of stress and joy behaviors, as well as body posture—used to assess dogs’ responses to conspecific emotional odors revealed significant differences between responses to the blank condition compared to conspecific emotional odors. Specifically, dogs exhibited fewer stress behaviors (e.g., ear flattening, lip licking) and more joy behaviors (e.g., play bows, play panting) following exposure to the blank condition, relative to both the joy and stress odor conditions. These findings suggest that exposure to conspecific emotional odors—regardless of emotional valence—may heighten stress and reduce joy behavioral responses in dogs relative to a neutral, nonsocial stimulus (e.g., a cotton pad with no odor collected). This pattern contrasts with previous studies involving human-derived odors, in which dogs exhibited more stress behaviors in response to human fear odors compared to happiness odors or the blank condition^2^. Postural data further show that emotionally salient conspecific odors elicit deviations from neutral behavioral states in dogs. When exposed to the blank condition, dogs displayed more upright postures and fewer prone postures than those exposed to either emotional odor. This pattern may indicate that the upright posture reflects a baseline or default state, while exposure to conspecific odors—both of which conveyed a kind of arousal in this study—promote a shift toward prone positioning. Although sitting also represents a deviation from an upright posture, no significant differences in sitting behavior were observed across odor conditions, likely due to its relatively low occurrence (upright: 169; sit: 20; prone: 94). While we found that measures of stress and joy behaviors, as well as posture, may serve as general indicators of exposure to social versus nonsocial (blank) stimuli, we did not observe these behavioral measures to be reliable indicators of emotional contagion, since there were no differences between exposure to stress versus joy odors. Although we found that dogs’ proximity to their owner and a stranger may suggest emotional contagion in response to conspecific emotional odors, we did not observe the behavioral mirroring that emotional contagion would predict to occur (e.g., as in D’Aniello et al.^2^): dogs did not display increased stress behaviors after exposure to the stress odor, nor increased joy behaviors after exposure to the joy odor. Our findings suggest that conspecific odors may influence dogs’ emotional states, but emotional contagion likely requires a more nuanced assessment of diverse behavioral cues, as its effects may not appear in a reliable manner. While we focused solely on measuring behavioral responses in this study, future research should incorporate physiological measures, such as heart rate, to further assess the presence and manifestation of emotional contagion in response to conspecific emotional odors.

Male and female dogs responded differently to conspecific emotional odor conditions, providing novel insights into the role of olfaction in canine social dynamics. Female dogs exposed to conspecific stress odors exhibited more stress behaviors, fewer joy behaviors, and greater proximity to their owners compared to male dogs, indicating a stronger and more aversive response to the stress odor. These findings may reflect a general female tendency to respond more strongly to conspecific stress odors, or—since the odor stimuli is from a female dog—they could suggest that dogs may be able to integrate both the sex and emotional content of the odor source when processing and responding to conspecific odors. Further research is needed to explore how males and females may differentially react to male-derived odors across different emotional states. Moreover, the small sample size within each sex per condition in this study may limit the generalizability of these findings. This study also revealed that male dogs showed greater proximity to strangers when exposed to the conspecific joy odor cognition, contrasting with D’Aniello et al.^40^, who reported that females in a human happiness odor condition displayed greater interest in strangers than males. This discrepancy, along with the observation that exposure to human emotional chemosignals did not yield sex-dependent behavioral responses related to the owner or stress^40^, but did so for conspecific odors in this study, underscores the potential differences in how dogs respond to emotional cues from human versus conspecific odors. These results offer a possible explanation for why conspecific joy odors may have elicited different responses compared to human joy odors^2^, as previously discussed.

Future research could broaden the scope of odor sources to improve the generalizability of findings regarding canine olfactory discrimination and emotional contagion, while also elucidating the chemical mechanisms enabling dogs to distinguish these signals. In the present study, odors were derived exclusively from a single, unfamiliar donor dog. To better understand dogs’ responses, subsequent investigations could explore reactions to composite odor stimuli pooled from multiple conspecifics. Given that our study involved a single donor for odor stimuli, we presented an identical odor in the habituation phase of the habituation-discrimination paradigm, whereas studies like Jardat et al.^24^ exposed subjects to same-category stimuli from different donors in the habituation phase. We chose to use a single donor to control for the potential influence of variables, such as donor identity and their demographic factors (e.g., age, sex, etc.), and thereby isolate emotional context as the only differentiating factor between stimuli. While using an identical odor presents some limitations—such as the possibility that dishabituation or discrimination might arise from sample identity rather than emotional context—our findings suggest that this was not the case. Had dogs discriminated based on sample identity rather than emotional context, we would have expected dishabituation and discrimination in all emotional conditions, which was not observed. Moreover, there were no systematic differences in the collection methods between the novel and habituated odors (i.e., across emotional context), indicating that emotional context, rather than sample identity, was the primary distinction between the novel and habituated odor. A future study incorporating stimuli from different dogs within the same emotional context across habituation phases could strengthen our current findings. Additionally, implementing a larger sample size of subject dogs interacting with these odors—especially for evaluating behavioral manifestations to conspecific emotional odors (*Part 2*)—would further improve the robustness of the results. Future studies could also assess whether emotional odors from familiar dogs reveal stronger emotional contagion effects, as existing literature indicates that familiarity can enhance social responses^52,53^.

Chemical analyses could be pursued to identify the specific volatile compounds that enable subjects to differentiate emotional odors in conspecifics, consistent with the concept of chemical fingerprints in emotional odors, as proposed by Semin et al.^54^. Identifying these chemical cues would help elucidate the mechanisms underlying dogs’ discrimination behavior and enhance the reliability of collected body odors in accurately reflecting the intended emotional state. Investigating the chemical signatures associated with joy odors specifically, and elucidating what dogs are detecting, could further validate that this emotional state can be readily detectable by dogs; currently, hypotheses regarding the chemical signatures dogs detect in positively valenced emotional odors have received limited attention. For negatively valenced emotions, the events designed to induce these emotions in dogs, both in the present study and in previous studies^19^, lasted only a few minutes (2–5), likely capturing the fast response biomarker (e.g., salivary alpha-amylase^55)^ but not the slower biomarker (e.g., cortisol, which peaks at 21–30 minutes^42)^. Future studies could devise sample collection methods targeting specific odor constituents in conspecific emotional odors (e.g., fast and slow response components in the stress odor) to better understand how these constituents may influence conspecific behavior differently and delineate the pathways triggering specific responses, thereby revealing the additional complexity of how dogs perceive and respond to conspecific emotional odors.

Our findings demonstrate that subject dogs were capable of distinguishing the emotional states of conspecifics and provide evidence that odors can mediate emotional transfer between dogs. These results contribute to a growing body of literature on canine emotional and olfactory communication^2,14,15,19,40^, extending previous research that primarily focused on the role of odors in interactions between dogs and humans. Specifically, our study highlights the role of odors in emotional communication among dogs, broadening the scope of how olfactory cues influence canine social dynamics. While previous research has found dogs’ ability to detect and respond to human emotional cues is not a shared trait across domesticated companion mammals and may have emerged through selection for cooperation during the domestication of dogs^39^, this study provides initial evidence that similar social abilities—like detecting and responding to emotional states in conspecific odors—may have existed in dog-to-dog interactions prior to domestication. These socio-cognitive skills for intraspecific communication could have provided the basis for domestication, enabling dogs to later apply these abilities to interspecific, human-directed communication. As such, this work contributes to our understanding of the processes that may have given rise to human-directed behaviors in dogs. Future studies using a wolf model to examine whether they can perceive emotional states in human and conspecific odors would help clarify whether detecting and responding to human chemosignals could be rooted in these foundational abilities. By demonstrating that dogs can communicate emotional states to conspecifics through olfactory cues in the absence of direct contact, this study also offers a new line of inquiry for future research to explicitly test how such scent-based signals may affect canine welfare in shared environments—such as dog shelters, parks, daycare centers, training facilities, and veterinary clinics—and to ultimately inform strategies that improve the care of dogs in these settings.

**Table 1:** Ethogram of behaviors categorized as indicative of stress or joy. Stress behaviors adapted from D’Aniello et al.^2^. * Denotes behaviors that were not observed in this study.

Behavioral categories	Description
Stress behaviors	Flattened ears, self-scratching, lip/nose licking (except after drinking), yawning, salivation*, paw lift*, cowering*, piloerection*, tail tucked*, lip retracting or grimacing*, vocalizing*, trembling*, pacing*
Joy behaviors	Play bow/slap, leaping on, relaxed open-mouth (“play face”), play panting, relaxed ears, tail wag (not low), leisurely exploring (dog > 1 body length from owner, box, and stranger), pouncing*, zoomies*

## Data Availability

The anonymized datasets collected in this study and the R code used for analysis are available on the corresponding Dryad Digital Repository, https://doi.org/10.5061/dryad.bzkh189pt, or upon request.
